# Exercise Interventions Incorporating Aerobic Components in Children and Adolescents with Acute Lymphoblastic Leukemia: A Systematic Review and Meta-Analysis

**DOI:** 10.3390/cancers18213454

**Published:** 2026-10-26

**Authors:** Joaquín González-Aroca, Álvaro Puelles-Diaz, Estefanny Castillo-Rivera, Claudia Gomila-Cisternas, Catalina Venegas-López, Jorge Olivares-Arancibia, Markel Rico-Gonzalez, José Francisco López-Gil, Javier Grau, Exal Garcia-Carrillo, Julio Plaza-Diaz

**Affiliations:** 1 Escuela de Kinesiología, Facultad de Salud, Universidad Santo Tomás, La Serena 1720236, Chile; jgonzalez180@santotomas.cl; 2 Escuela de Tecnología Médica, Facultad de Ciencias de la Salud, Universidad del Alba, La Serena 1720236, Chile; 3 Carrera de Medicina, Facultad de Ciencias de la Salud, Universidad del Alba, La Serena 1720236, Chile; apuelles@udalba.cl; 4 Department of Kinesiology, University of La Serena, La Serena 1720236, Chile; estefanny.castillo@userena.cl (E.C.-R.); claudia.gomila@userena.cl (C.G.-C.); catalina.venegasl@userena.cl (C.V.-L.); javier.grauu@userena.cl (J.G.); 5 AFySE Group, Research in Physical Activity and School Health, School of Physical Education, Faculty of Education, Universidad de las Américas, Santiago 7500975, Chile; jorge.olivares.ar@gmail.com; 6 Department of Didactics of Musical, Plastic and Corporal Expression, University of the Basque Country, EHU, 48940 Leioa, Spain; markel.rico@ehu.eus; 7 School of Medicine, Universidad Espíritu Santo, Samborondón 092301, Ecuador; 8 Faculty of Health Sciences, Universidad Autónoma de Chile, Temuco 4810101, Chile; 9 Department of Physical Activity Sciences, Faculty of Education Sciences, Universidad Católica del Maule, Talca 3480112, Chile; exal.garcia@gmail.com; 10 School of Education, Faculty of Human Sciences, Universidad Bernardo O’Higgins, Santiago 8370993, Chile; 11 Departament de Bioquímica i Biotecnologia, ANUT-DSM (Alimentació, Nutrició, Desenvolupament i Salut Mental), Universitat Rovira i Virgili, 43201 Reus, Spain; julioramon.plaza@urv.cat; 12 Institut de Recerca Biomèdica Catalunya Sud, Hospital Universitari Sant Joan de Reus, 43204 Reus, Spain; 13 Biomedical Research Networking Center for Physiopathology of Obesity and Nutrition (CIBERObn), Institute of Health Carlos III, 28029 Madrid, Spain

**Keywords:** acute lymphoblastic leukemia, pediatric oncology, rehabilitation, physical activity, aerobic exercise, quality of life, systematic review, meta-analysis

## Abstract

**Background**: Acute lymphoblastic leukemia (ALL) is the most common childhood cancer. Despite substantial improvements in survival, children and adolescents with ALL frequently experience treatment-related impairments in physical function, mobility, and quality of life. Exercise interventions incorporating aerobic components have been proposed as supportive rehabilitation strategies to mitigate these sequelae. **Objective**: This study aimed to evaluate the effects of exercise interventions containing aerobic components as adjuncts to standard treatment on quality of life and related functional outcomes in children and adolescents with ALL. **Methods**: This systematic review was conducted in accordance with the Preferred Reporting Items for Systematic Reviews and Meta-Analyses (PRISMA) guidelines and prospectively registered in PROSPERO (CRD42024581010). MEDLINE (via PubMed), Web of Science, and Scopus were searched from inception to April 2026. The Cochrane Central Register of Controlled Trials (CENTRAL), ClinicalTrials.gov, and the World Health Organization (WHO) International Clinical Trials Registry Platform (WHO ICTRP) were additionally searched on 3 October 2026. Randomized controlled trials evaluating structured exercise interventions with an aerobic component in children and adolescents with ALL were included. The primary outcome was quality of life. Secondary outcomes included functional mobility, functional capacity, and related physical performance outcomes. Random-effects meta-analyses were performed when appropriate, and certainty of evidence was assessed using the Grading of Recommendations Assessment, Development and Evaluation (GRADE) approach. **Results**: Five randomized controlled trials involving 254 participants met the inclusion criteria. Interventions consisted of supervised or home-based exercise programs incorporating aerobic activities in combination with other rehabilitation modalities. Meta-analysis suggested improvements in functional mobility and functional capacity compared with control interventions (low-certainty evidence). Quality-of-life findings were inconsistent across studies and were not suitable for quantitative pooling because of heterogeneity in instruments, reporting methods, and outcome domains; the certainty of evidence was rated as very low. No serious exercise-related adverse events were reported, although safety monitoring was inconsistently described. **Conclusions**: The effects of exercise interventions containing aerobic components on quality of life in children and adolescents with ALL remain very uncertain. Low-certainty evidence suggests that these interventions may improve functional mobility and functional capacity. Additional adequately powered, methodologically rigorous randomized controlled trials with standardized outcome measures, predefined adverse-event reporting, and comprehensive safety monitoring are required to establish the effectiveness and safety of these interventions in pediatric oncology rehabilitation.

## Simple Summary

Children and adolescents with acute lymphoblastic leukemia may experience difficulties with physical activity, mobility, and quality of life during and after cancer treatment. We reviewed five randomized studies involving 254 participants to examine whether exercise programs that include aerobic activities, such as walking or cycling, can support their recovery. These programs may improve mobility, particularly the ability to go up and down stairs, but their effects on quality of life remain very uncertain. Confidence in the findings was limited by small studies, differences between exercise programs, and weaknesses in study methods. No serious exercise-related adverse events were reported, although safety monitoring was often poorly described. These findings suggest a potential role for exercise in rehabilitation, while highlighting the need for larger, well-designed studies to establish its benefits and safety and guide care for children and adolescents with this disease.

## 1. Introduction

Acute lymphoblastic leukemia (ALL), also referred to as acute lymphocytic or acute lymphoid leukemia, is a malignant hematologic neoplasm characterized by the uncontrolled proliferation of immature lymphoid precursors that infiltrate the bone marrow [1]. Its rapid clinical progression reflects the aggressive expansion of these precursor cells and the consequent suppression of normal hematopoiesis [2].

Clinically, ALL may present with constitutional and systemic symptoms such as weakness, fatigue, musculoskeletal pain, and dyspnea, along with signs including fever, night sweats, easy bruising, spontaneous bleeding, unexplained weight loss, anorexia, and lymphadenopathy [3]. ALL is the most common childhood cancer. Survival has improved markedly over recent decades, with 5-year survival reaching 92.3% among children diagnosed in the United States during 2014–2020 [4]. Despite therapeutic advances and improved remission rates, many pediatric ALL survivors exhibit significant physical deconditioning as a sequela of standard treatment modalities, including chemotherapy, radiotherapy, hematopoietic stem cell transplantation, and targeted therapies, which may result in reduced functional capacity and diminished quality of life [5,6].

To address these outcomes, various pharmacological and non-pharmacological strategies have been proposed, including symptom-targeted medications (e.g., analgesics, antiemetics, corticosteroid dose adjustments, and agents addressing fatigue or mood disturbances) alongside supportive rehabilitation approaches. Among the non-pharmacological interventions, supervised aerobic exercise has garnered increasing attention as a potential adjunctive strategy to standard treatment. This form of exercise involves sustained rhythmic movement of large muscle groups at low-to-moderate intensity, eliciting widespread physiological benefits [7]. From a cardiovascular perspective, exercise training has shown benefits in selected measures of cardiorespiratory fitness, muscle strength, and flexibility in children receiving treatment for ALL; however, findings are inconsistent across assessments, and the supporting evidence is limited and of low quality [5]. Additionally, evidence from other pediatric oncology populations suggests potential neurocognitive benefits of exercise. In a controlled clinical trial involving pediatric brain tumor survivors treated with cranial radiation, exercise training improved white matter structure, hippocampal volume, and reaction time [8]. 

Previous systematic reviews [6,9] have suggested that exercise interventions may benefit children and adolescents with ALL by improving physical performance, functional mobility, and other health-related outcomes. However, the available evidence remains limited by the small number of randomized trials, heterogeneity in intervention characteristics, and variation in outcome measures. Furthermore, additional randomized controlled trials have been published since earlier reviews were conducted. Therefore, an updated systematic review was undertaken to synthesize the current evidence regarding exercise interventions containing aerobic components and their effects on quality of life and related functional outcomes in pediatric patients with ALL.

Based on the available evidence in other oncological and pediatric populations, we hypothesized that exercise interventions containing aerobic components, when implemented as a complementary intervention to standard treatment, would have a positive effect on quality of life and related functional outcomes in pediatric patients with acute lymphoblastic leukemia compared with standard care alone. In this context, the present study aims to synthesize the available evidence on aerobic exercise as a complementary intervention to standard therapy in pediatric patients with ALL, compared with standard care alone, with particular emphasis on quality of life and related functional outcomes.

## 2. Materials and Methods

This systematic review was conducted following the PRISMA (Preferred Reporting Items for Systematic Reviews and Meta-Analyses) [10] guidelines and the Cochrane handbook for systematic reviews of interventions [11]. The protocol was prospectively registered in the International Prospective Register of Systematic Reviews (PROSPERO; ID: CRD42024581010).

### 2.1. Criteria for Considering Studies for This Review

#### 2.1.1. Types of Studies

We included randomized controlled trials (RCTs), regardless of language, publication year, or publication status. Observational studies, non-randomized and quasi-randomized trials, and crossover trials were excluded.

#### 2.1.2. Types of Participants

Eligible studies involved children and adolescents from birth to 18 years of age diagnosed with ALL, who were receiving or had completed standard treatment, including chemotherapy (regardless of the type), radiotherapy, stem cell transplantation, and/or targeted therapy. We excluded studies involving participants with severe respiratory diseases (e.g., pulmonary embolism, interstitial lung disease, pulmonary hypertension, respiratory failure, pulmonary fibrosis, or cystic fibrosis), congenital cardiovascular or neurological disorders, other cancer types, patients undergoing surgical interventions such as lobectomy, and individuals with formal contraindications to physical exercise.

#### 2.1.3. Types of Interventions

We included RCTs evaluating structured exercise interventions that incorporated a prespecified aerobic exercise component during or after treatment for ALL. Eligible interventions could consist exclusively of aerobic exercise or combine aerobic exercise with strengthening, flexibility, range of motion, motor, behavioral, or other rehabilitation components. Because most eligible interventions were multimodal, inclusion in this review indicates the presence of an aerobic component and does not permit attribution of intervention effects specifically to aerobic exercise.

#### 2.1.4. Types of Outcome Measures

Primary outcome

The primary outcome was health-related quality of life. Relevant instruments included the Pediatric Quality of Life Inventory (PedsQL) 4.0 Generic Core Scales and the Pediatric Quality of Life Inventory 3.0 Cancer Module, for which reliability and validity have been examined in children with cancer [12]. Child self-reports and parent-proxy reports were considered separately.

Secondary outcomes

Functional exercise capacity/endurance included the six-minute walk test (6MWT) and the nine-minute walk/run–walk tests. Pediatric reliability and validity evidence for the six-minute walk test was provided by Li et al. [13], while the interpretation of the nine-minute tests was supported by their use and descriptions in the included ALL studies [14,15]. Functional mobility included the Timed Up and Go (TUG) and Timed Up and Down Stairs (TUDS) tests. The classification of TUG was supported by research in children with ALL [16], while pediatric reliability and validity evidence for TUDS was provided by Zaino et al. [17]. Cardiorespiratory fitness was considered separately from functional exercise capacity/endurance and included directly measured peak oxygen uptake (VO_2_peak). Functional status, depression, and anxiety were additional eligible outcome domains. For instruments assessing these domains, the age range and population supporting their measurement properties were considered explicitly; evidence obtained in adult populations was not assumed to establish pediatric validity.

### 2.2. Search Methods for Identification of Studies

Relevant trials were searched from inception to April 2026 in MEDLINE (via PubMed), Web of Science, and Scopus. To improve sensitivity and identify ongoing, unpublished, or otherwise potentially eligible studies, the search was subsequently expanded to additional trial sources. The Cochrane Central Register of Controlled Trials (CENTRAL), ClinicalTrials.gov, and the World Health Organization International Clinical Trials Registry Platform (WHO ICTRP) were additionally searched (3 October 2026).

For bibliographic databases, the search strategy was developed using Medical Subject Headings (MeSH), where applicable, together with relevant free-text terms. Registry searches were adapted to the search structure of ClinicalTrials.gov and WHO ICTRP. Search terms combined concepts related to acute lymphoblastic leukemia with exercise- and rehabilitation-related terms, including “exercise”, “physical activity”, “rehabilitation”, “physical therapy”, and “aerobic exercise”.

The supplementary search identified no records in CENTRAL, 112 registry records in ClinicalTrials.gov, and 28 registry records in WHO ICTRP. All registry records were screened using the same predefined eligibility criteria applied to bibliographic records. None resulted in an additional eligible randomized controlled trial for inclusion in the review. We did not perform a separate search specifically for adverse events associated with exercise interventions. Only adverse events reported in the included studies were considered.

As an example, the search strategy used for MEDLINE (via PubMed) was as follows:

(“Exercise” [Title/Abstract] OR “Acute Exercise” [Title/Abstract] OR “Physical exercise” [Title/Abstract] OR “Home exercise” [Title/Abstract] OR “Physical activity” [Title/Abstract] OR “Sport” [Title/Abstract] OR “Sports” [Title/Abstract] OR “Rehabilitation exercise” [Title/Abstract] OR “Training” [Title/Abstract] OR “Exercise Training” [Title/Abstract] OR “Physical therapy” [Title/Abstract] OR “Exercise therapy” [Title/Abstract] OR “Aerobic exercise” [Title/Abstract]) AND (“Acute Lymphoblastic Leukemia” [Title/Abstract] OR “Precursor Cell Lymphoblastic Leukemia-Lymphoma” [Title/Abstract] OR “acute lymphocytic leukemia” [Title/Abstract] OR “Acute lymphoid leukemia” [Title/Abstract] OR “LLA” [Title/Abstract] OR “Acute leukemia” [Title/Abstract] OR “Leukemia” [Title/Abstract] OR “leukaemia” [Title/Abstract] OR “acute leukaemia” [Title/Abstract]).

Reference lists of included trials and relevant systematic reviews were also screened for additional eligible studies.

CENTRAL search strategy.

#1 MeSH descriptor: [Precursor Cell Lymphoblastic Leukemia–Lymphoma] explode all trees

#2

(“acute lymphoblastic leukemia” OR

“acute lymphoblastic leukaemia” OR

“acute lymphocytic leukemia” OR

“acute lymphocytic leukaemia” OR

“acute lymphoid leukemia” OR

“acute lymphoid leukaemia” OR

“acute leukemia” OR

“acute leukaemia”):ti,ab,kw



#3 #1 OR #2

#4 MeSH descriptor: [Exercise] explode all trees

#5 MeSH descriptor: [Exercise Therapy] explode all trees

#6 MeSH descriptor: [Physical Therapy Modalities] explode all trees



#7

(exercis* OR

“physical activ*” OR

“physical exercise” OR

“exercise training” OR

“aerobic exercise” OR

“aerobic training” OR

“endurance training” OR

“home exercise” OR

sport* OR

rehabilitat* OR

physiotherap* OR

“physical therap*” OR

“exercise therap*” OR

training OR

walking OR

cycling):ti,ab,kw



#8 #4 OR #5 OR #6 OR #7

#9 #3 AND #8



**Trial registry searches**

To identify ongoing, unpublished, or otherwise potentially eligible trials, additional searches were conducted in ClinicalTrials.gov and the World Health Organization International Clinical Trials Registry Platform (WHO ICTRP). Searches were performed using combinations of terms related to acute lymphoblastic leukemia and exercise or rehabilitation. For ClinicalTrials.gov, the condition field was searched using “acute lymphoblastic leukemia”, and the following terms were entered separately in the “Other terms” field: “exercise”, “physical activity”, “rehabilitation”, “physical therapy”, and “aerobic”. For WHO ICTRP, searches combined “acute lymphoblastic leukemia” with each of the following terms: “exercise”, “physical activity”, “rehabilitation”, “physical therapy”, and “aerobic”. Alternative disease terminology, including “acute lymphoblastic leukaemia” and “acute lymphocytic leukemia”, was additionally searched when necessary to maximize sensitivity. No restrictions were applied according to recruitment status or publication status. Potentially relevant registry records were screened according to the same eligibility criteria applied to bibliographic records, and duplicate registrations were identified and removed before study selection.

### 2.3. Data Management

Duplicate records were removed using the free version of Covidence software (Veritas Health Innovation, Melbourne, Australia). Remaining references were exported to Rayyan (Rayyan Systems Inc., Cambridge, MA, USA), an open-access platform used for screening. All citations and study metadata were managed with the Zotero reference manager (Version: 9.0.6; Corporation for Digital Scholarship, Vienna, VA, USA).

### 2.4. Study Selection and Data Extraction

Two reviewers (C.G.-C. and E.C.-R.) independently and blindly screened the records. Any disagreements were resolved by a third reviewer (C.V.-L.). After deduplication, all titles and abstracts were screened independently to determine eligibility based on predefined inclusion and exclusion criteria. Studies were classified as “eligible”, “not eligible”, or “potentially eligible”. Full-text screening was then conducted independently by the same reviewers (C.G.-C. and E.C.-R.), with reasons for exclusion documented. Trial-registry records identified through ClinicalTrials.gov and WHO ICTRP were screened using the same predefined eligibility criteria as bibliographic records, and linked publications or posted results were reviewed when available. The PRISMA flow diagram was constructed on the basis of the final study set.

For data extraction, two reviewers (C.G.-C. and C.V.-L.) independently extracted data using a standardized form. Discrepancies were resolved by consensus or, if necessary, with input from a third author (E.C.-R.).

### 2.5. Assessment of Risk of Bias in Included Studies

Two reviewers (E.C.-R. and C.V.-L.) independently assessed the risk of bias using the Cochrane Handbook for Systematic Reviews of Interventions criteria, RoB 2 [18]. Any disagreements were resolved by a third author (C.G.-C.). The domains assessed included the following:

Bias from the randomization process;

Bias due to deviations from intended interventions;

Bias from missing outcome data;

Bias in outcome measurements;

Bias in the selection of reported results.

The RoB 2 Excel tool was used to complete the bias assessments, following its standard algorithm.

### 2.6. Synthesis Methods

A meta-analysis was performed when at least two studies reported comparable outcomes and sufficient numerical data were available. Due to the limited number of included studies and expected clinical heterogeneity in exercise protocols, random-effects models were used for all quantitative analyses.

Two outcome domains were considered suitable for quantitative synthesis:

- Functional mobility, which was assessed using the TUDS test.
- Functional capacity, which was assessed using field-based exercise performance tests, including the 6MWT, 9 min walk test, and 9 min run–walk test.

For functional mobility, pooled effects were calculated using mean differences (MDs) with 95% confidence intervals (CIs), as all studies reported the outcome using the same measurement scale (seconds).

Functional exercise capacity was synthesized using standardized mean differences (SMDs) calculated as Hedges’ g, incorporating a correction for small-sample bias. The six-minute walk, nine-minute walk, and nine-minute run–walk tests were grouped because they assess sustained locomotor performance and endurance. Differences in test duration, pacing, and permitted locomotor activity were acknowledged as potential sources of clinical heterogeneity.

The quantitative synthesis was restricted to post-intervention assessments in the medium term (3–6 months): 3 months for Tanir and Kuguoglu [14] and Elnaggar et al. [19] and 4 months for Marchese et al. [15]. Effect sizes were calculated using post-intervention means, standard deviations, and the numbers of participants contributing to the assessment in each group. Endpoint values were used consistently; endpoint and change-score SMDs were not combined. Each trial contributed one independent between-group estimate, avoiding double-counting across repeated assessments. The contributing trials randomized individual participants; therefore, adjustment for cluster randomization was not required. These analyses estimated unadjusted post-intervention differences rather than baseline-adjusted or group-by-time effects.

Effects were pooled using an inverse-variance random-effects model, with between-study variance estimated by restricted maximum likelihood (REML). Results were reported with 95% confidence intervals, and statistical heterogeneity was assessed using I^2^ and τ^2^. Positive Hedges’ g values favored exercise. A *post-hoc* sensitivity analysis excluded Marchese et al. [15] to examine the influence of including a test that permitted running.

Quality-of-life outcomes were not quantitatively synthesized due to heterogeneity in the assessment instruments, reporting methods, and outcome domains. Studies used different versions of the PedsQL, including both generic and cancer-specific modules, and several studies reported only subscale scores or insufficient data for effect size calculation. Therefore, quality-of-life findings were synthesized narratively.

Statistical heterogeneity was assessed through visual inspection of forest plots and quantified using the I^2^ statistic, following recommendations from the Cochrane Handbook for Systematic Reviews of Interventions. Heterogeneity was interpreted according to conventional thresholds, whereby I^2^ values of 0–40% were considered potentially unimportant, 30–60% were indicative of moderate heterogeneity, 50–90% represented substantial heterogeneity, and 75–100% represented considerable heterogeneity [20].

All statistical analyses were performed using Stata (Stata BE 17, StataCorp, College Station, TX, USA). Statistical significance was defined as *p* < 0.05.

For outcomes synthesized using SMDs, effect sizes were interpreted according to Cohen’s [21] conventional thresholds, where 0.2 represents a small effect, 0.5 a moderate effect, and 0.8 a large effect. These thresholds were used to facilitate interpretation of the magnitude of pooled effects across studies using different measurement scales.

### 2.7. Certainty of the Evidence Assessment

We constructed a summary of findings (SoF) table using the Grading of Recommendations Assessment, Development and Evaluation (GRADE) [22] approach to summarize the certainty of the evidence and the magnitude of effects for the main outcomes of this review. The SoF table included functional mobility, functional capacity, and quality of life. For outcomes synthesized quantitatively, we reported pooled effect estimates as MDs or SMDs with corresponding 95% CIs, together with the number of studies and participants contributing to each outcome. For outcomes that could not be pooled quantitatively, findings were summarized narratively.

The certainty of the evidence for each outcome was assessed using the GRADE approach, considering five domains: risk of bias, indirectness, inconsistency, imprecision, and publication bias. Evidence derived from randomized controlled trials was initially rated as high certainty and subsequently downgraded when concerns were identified in one or more domains. Evidence was downgraded by one level when concerns were considered serious and by two levels when concerns were considered very serious. The final certainty ratings were classified as high, moderate, low, or very low.

We assessed each outcome individually and gave it a high, moderate, low or very low rating for the certainty of evidence, using the following definitions.

1. High certainty: We are very confident that the true effect lies close to that of the estimate of the effect.

2. Moderate certainty: We are moderately confident in the effect estimate: the true effect is likely to be close to the estimate of the effect, but there is a possibility that it is substantially different.

3. Low certainty: Our confidence in the effect estimate is limited: the true effect may be substantially different from the estimate of the effect.

4. Very low certainty: We have very little confidence in the effect estimate: the true effect is likely to be substantially different from the estimate of effect. Where we downgraded the certainty of the evidence, we described our reasons and the supporting information for our decisions in the footnotes of the summary of findings table. If it had been necessary, we would have contacted the study authors for clarification of details to allow judgements to be made.

## 3. Results

**Figure 1:**
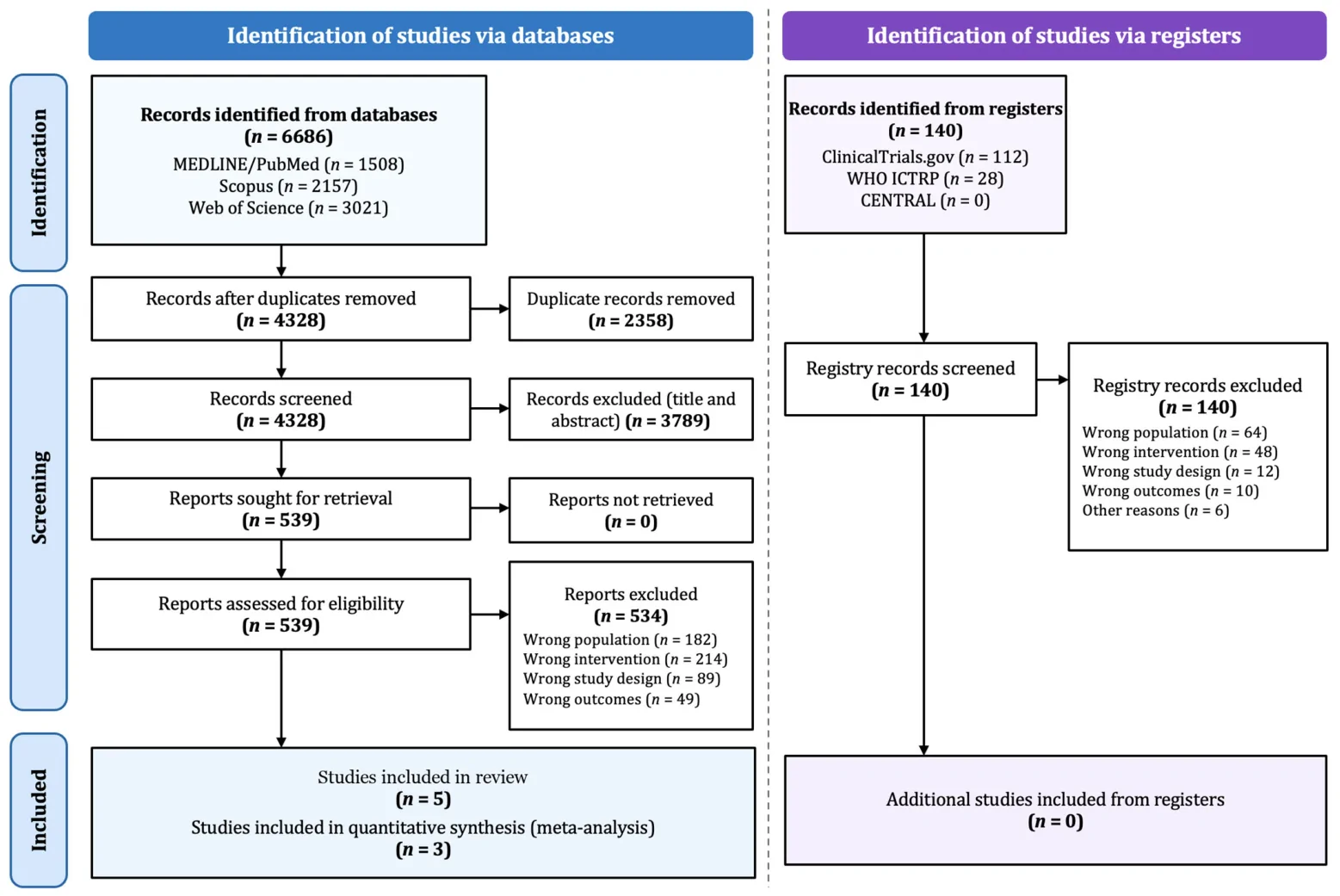
PRISMA 2020 flow diagram of the literature search and study-selection process (blue: database searches; purple: supplementary register searches). The original bibliographic search included MEDLINE/PubMed, Scopus, and Web of Science. Supplementary searches of CENTRAL, ClinicalTrials.gov, and the WHO International Clinical Trials Registry Platform (WHO ICTRP) were conducted subsequently. The diagram distinguishes records screened, reports sought for retrieval, reports not retrieved, reports assessed for eligibility, and studies included. The registry searches identified 140 records, none of which resulted in an additional eligible randomized controlled trial.

As shown in Figure 1, a total of 6686 records were identified through database searches, including PubMed (*n* = 1508), Scopus (*n* = 2157), and Web of Science (*n* = 3021). After removal of 2358 duplicate records, 4328 records underwent title and abstract screening, of which 3789 were excluded. The remaining 539 reports were sought for retrieval and assessed for full-text eligibility. Of these, 534 were excluded for the reasons shown in Figure 1, leaving five randomized controlled trials for inclusion in the review [14,15,19,23,24]. Supplementary searches identified no records in CENTRAL, 112 registry records in ClinicalTrials.gov, and 28 registry records in WHO ICTRP. All 140 registry records were screened against the predefined eligibility criteria, and none resulted in an additional eligible study.

### 3.1. Characteristics of the Included Studies

The characteristics of the included studies are summarized in Table 1. A total of 254 participants were initially included across five randomized controlled trials. Sex-distribution data were available for 253 participants, of whom 161 were male (63.6%). The mean age reported across studies ranged from 7.7 to 13.8 years, although variability existed according to treatment phase and eligibility criteria. Participants included children undergoing active chemotherapy, patients receiving maintenance therapy, children in remission, and survivors assessed after treatment completion. All studies recruited participants who were physically able to engage in exercise-based interventions.

All included studies implemented structured exercise-based interventions with aerobic components. Elnaggar et al. [19] used a supervised dose-graded aerobic exercise program combined with traditional rehabilitation, whereas Khodashenas et al. [23] implemented a 12-week aerobic exercise program including walking, running, and play activities. Similarly, Tanir and Kuguoglu [14] applied a home-based intervention consisting of range-of-motion exercises, muscle strengthening, and aerobic activities such as walking, cycling, rope jumping, dancing, and slow running. Marchese et al. [15] administered an individualized physical therapy program including stretching, strengthening, aerobic exercise, and home-based exercises. Cox et al. [24] evaluated a long-term intervention initiated within 10 days of diagnosis and continued throughout treatment, combining motivational support with physical therapy exercises targeting strength, range of motion, motor skills, endurance, and physical activity promotion. The assessed outcomes mainly included physical performance, functional mobility, cardiopulmonary fitness, muscle strength, and quality of life. Quality of life was commonly evaluated using the PedsQL versions 3.0 and/or 4.0. Functional and physical performance outcomes included the 6MWT, TUDS, TUG, shuttle run tests, range of motion, and muscle strength assessments. Additionally, Elnaggar et al. [19] evaluated cardiopulmonary fitness parameters such as VO_2_peak, while Tanir and Kuguoglu [14] also assessed hematological variables including hemoglobin and hematocrit levels.

### 3.2. Risk of Bias ofIncluded Studies

**Figure 2:**
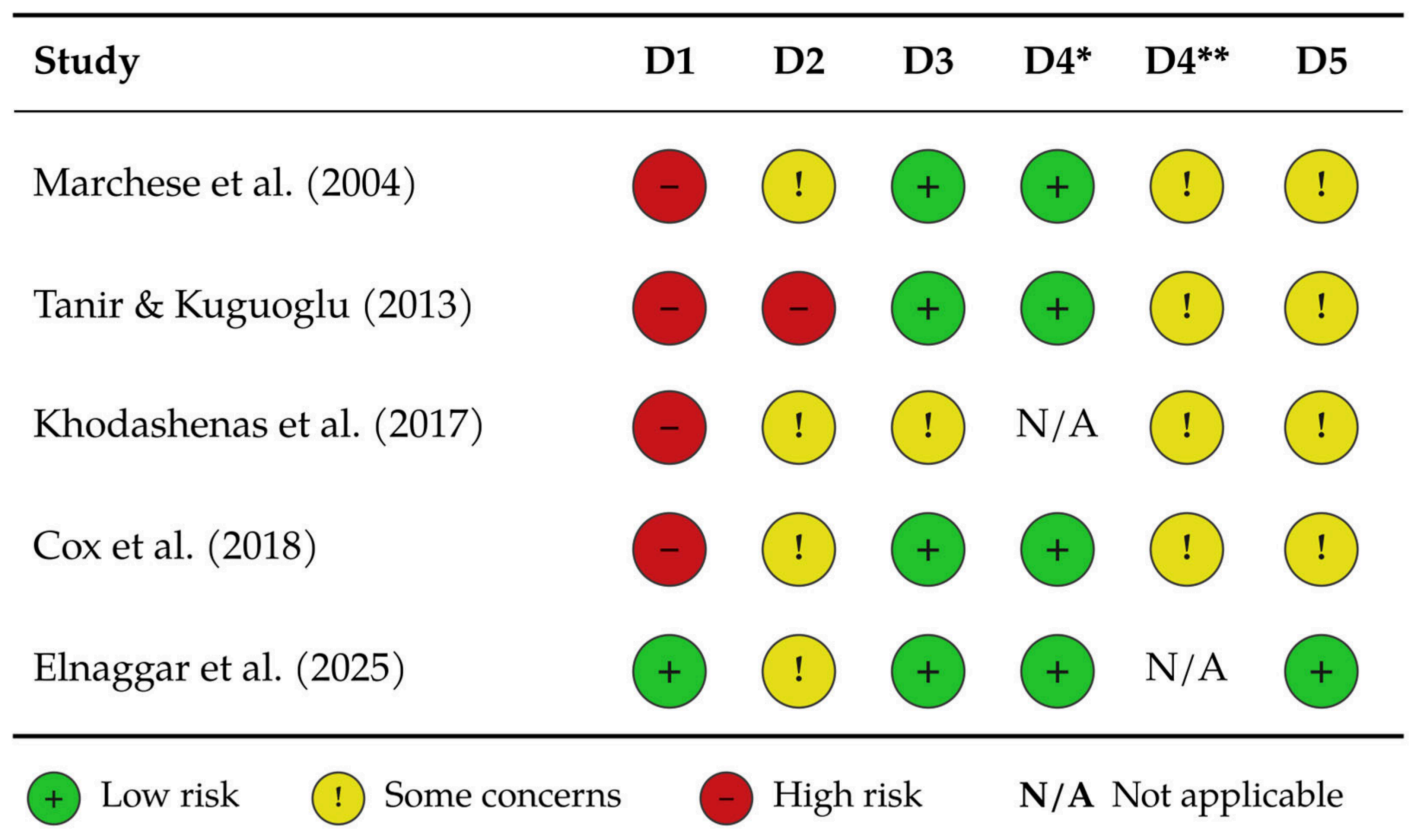
Risk-of-bias judgements for each included trial, assessed with the Cochrane risk-of-bias tool for randomized trials (RoB 2). **Abbreviations:** D1, randomization process; D2, deviations from intended interventions; D3, missing outcome data; D4, outcome measurement; D5, selection of the reported result; *, objective outcomes; **, subjective outcomes. Marchese et al. (2004) [15]; Tanir and Kuguoglu (2013) [14]; Khodashenas et al. (2017) [23]; Cox et al. (2018) [24]; Elnaggar et al. (2025) [19].

Figure 2 presents the individual risk of bias assessments for the included studies. The most common methodological limitations were related to the randomization process and deviations from intended interventions.

Four studies showed high risk of bias in the randomization domain due to insufficient reporting of sequence generation and allocation concealment procedures. In exercise-based interventions, blinding of participants and personnel was generally not feasible, contributing to concerns regarding deviations from intended interventions. Missing outcome data were generally well addressed across studies, with most trials judged as low risk in this domain. The outcome measurement for objective physical performance outcomes was generally judged as low risk of bias due to the use of standardized functional tests. In contrast, studies assessing subjective quality-of-life outcomes using self-reported or parent-reported instruments were judged as presenting some concerns because blinding procedures were poorly described.

### 3.3. Outcomes

See Table 2 for a summary of the findings.

#### 3.3.1. Functional Mobility

**Figure 3:**
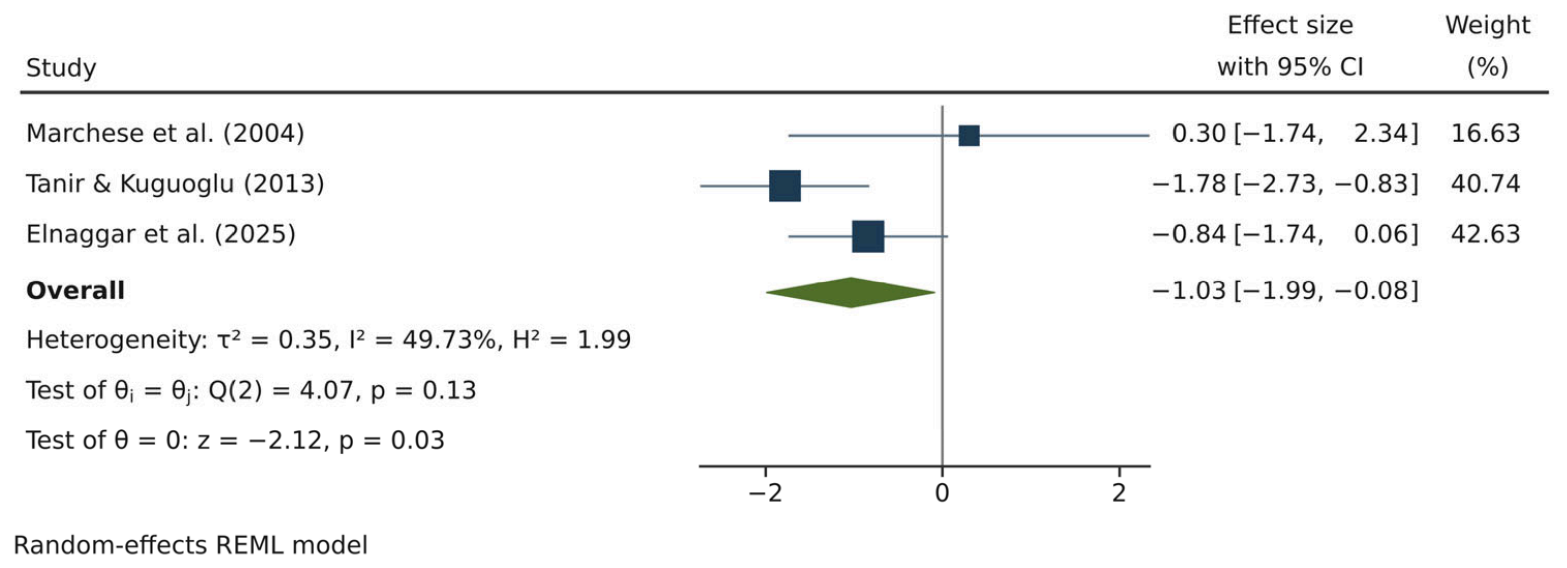
Forest plot of the effect of exercise interventions on functional mobility (Timed Up and Down Stairs test; mean difference in seconds; random-effects restricted maximum likelihood (REML) model). Negative values favor exercise. Marchese et al. (2004) [15]; Tanir and Kuguoglu (2013) [14]; Elnaggar et al. (2025) [19].

Three studies [14,15,19] (n = 126) reported functional mobility outcomes using the TUDS test. Pooled analysis showed that exercise interventions were associated with improved TUDS performance compared with control interventions (MD = −1.03 s, 95% CI −1.99 to −0.08; I^2^ = 49.7%; Figure 3). The certainty of the evidence was rated as low due to concerns regarding risk of bias and imprecision.

#### 3.3.2. Functional Exercise Capacity/Endurance

**Figure 4:**
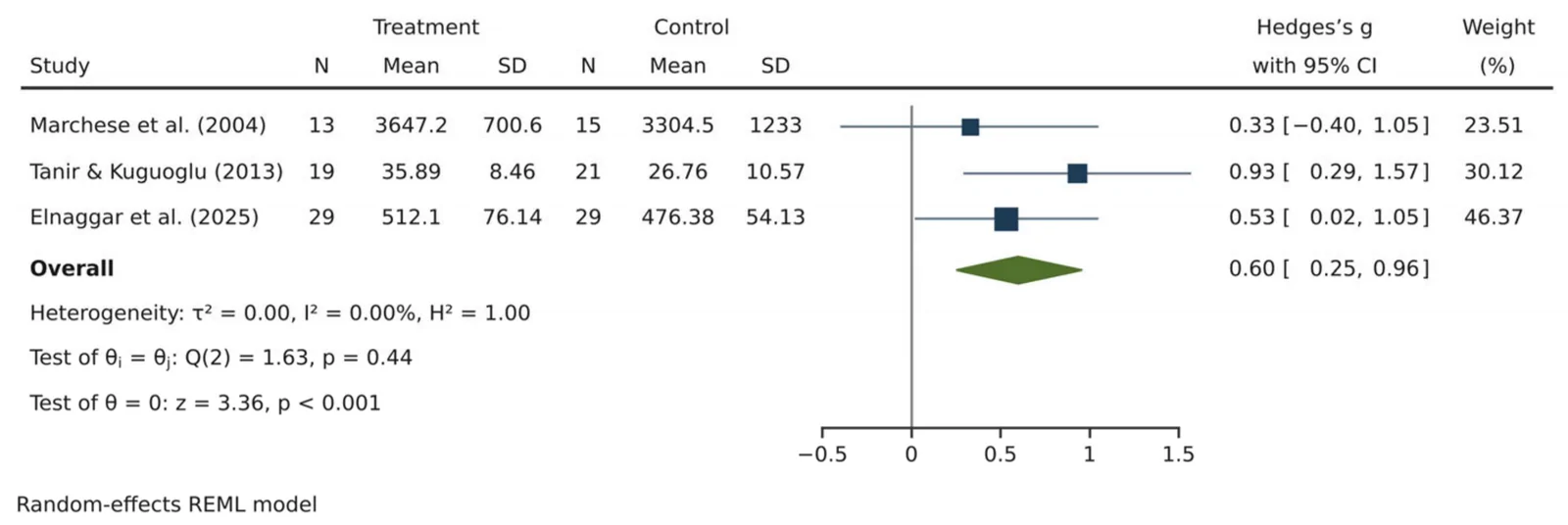
Forest plot of functional exercise capacity/endurance at 3–4 months (six-minute walk, nine-minute walk, and nine-minute run–walk tests; Hedges’ g; random-effects restricted maximum likelihood [REML] model). Positive values favor exercise. Marchese et al. (2004) [15]; Tanir and Kuguoglu (2013) [14]; Elnaggar et al. (2025) [19].

Three randomized controlled trials involving 126 participants contributed to the meta-analysis of functional exercise capacity/endurance at the medium term (3–6 months). The pooled post-intervention effect favored exercise interventions (Hedges’ g = 0.60, 95% CI 0.25–0.96; *p* < 0.001; Figure 4). No statistical heterogeneity was detected (I^2^ = 0%), although the small number of studies limits the assessment of heterogeneity. In the sensitivity analysis, excluding Marchese et al. [15], who used a nine-minute run–walk test, the pooled effect remained favorable (two trials with 98 participants; Hedges’ g = 0.69; 95% CI 0.29–1.09; *p* < 0.001; I^2^ = 0%; Supplementary Figure S1). The direction of the finding was therefore maintained when the analysis was restricted to walking tests. Cox et al. [24] reported six-minute walk distances at approximately 135 weeks of 477.51 m (standard deviation [SD] 145.65) in the intervention group and 515.69 m (SD 71.97) in the usual-care group. These findings were considered separately because of the substantially different assessment period and the absence of clearly reported outcome-specific sample sizes.

The certainty of the evidence was rated as low because of serious concerns regarding risk of bias and imprecision.

#### 3.3.3. Quality of Life

Four studies evaluated quality of life [14,15,23,24]. Khodashenas et al. [23], Tanir and Kuguoglu [14], and Marchese et al. [15] used different versions of the PedsQL. Cox et al. [24] used the Child Health Questionnaire (CHQ), not the PedsQL. Individual studies reported improvements in selected quality-of-life domains, including worry, cognitive problems, pain and hurt (reported by Khodashenas et al. [23] as “pain and injury”), and treatment-related concerns. However, findings were inconsistent across studies, and improvements were not observed across all domains. The certainty of the evidence was rated as very low.

#### 3.3.4. Adverse Events

No serious exercise-related adverse events were reported in the included studies; however, adverse event monitoring was poorly described or not explicitly reported in several trials.

#### 3.3.5. Functional Status, Depression, and Anxiety

No included trial reported functional status, depression, or anxiety.

## 4. Discussion

This systematic review synthesized randomized evidence on exercise interventions incorporating an aerobic component as adjuncts to standard therapy in children and adolescents with ALL. Five randomized controlled trials involving 254 participants met the inclusion criteria. Evidence regarding the primary outcome, quality of life, was very uncertain because findings were inconsistent across studies and were based on heterogeneous instruments and reporting approaches. For the secondary outcomes, low-certainty evidence suggests that exercise interventions containing aerobic components may improve functional mobility and functional exercise capacity/endurance at medium-term assessments. These findings should be interpreted cautiously because the available trials were small and methodologically heterogeneous, and because the observed effects cannot be attributed specifically to the aerobic component of predominantly multimodal interventions.

Low-certainty evidence from three randomized controlled trials suggests that exercise interventions may improve functional mobility, as reflected by better performance on the TUDS test. Functional mobility is an important component of daily functioning in children with ALL, as treatment-related impairments may affect balance, coordination, muscle strength, and the ability to perform routine physical activities. The observed improvement suggests that exercise interventions may help mitigate some of these treatment-related functional limitations.

Low-certainty evidence also suggests that these interventions may improve functional exercise capacity/endurance. The pooled analysis of three trials favored exercise interventions (Hedges’ g = 0.60; 95% CI 0.25–0.96), and the direction of the finding was maintained in a sensitivity analysis excluding the nine-minute run–walk test. Although no statistical heterogeneity was detected, the small number of trials limits its assessment, and differences in treatment phases, intervention characteristics, and test protocols remain relevant to interpretation. Both pooled analyses reflect assessments at the medium term (3–6 months). These findings should be interpreted cautiously because of the low certainty of evidence, and the benefits cannot be attributed specifically to the aerobic component of predominantly multimodal interventions.

Evidence regarding health-related quality of life was also very uncertain. Although some studies reported improvements in selected domains, including pain and hurt, cognitive problems, worry, and treatment-related concerns, findings were inconsistent across studies and improvements were not observed across all domains. Some improvements were reported primarily by parents rather than by children themselves, highlighting differences between proxy and self-reported outcomes. These reports should therefore be interpreted separately.

Although no serious exercise-related adverse events were reported, safety outcomes were inconsistently assessed and reported across studies. Importantly, none of the included trials systematically monitored potential exercise-related risks relevant to pediatric ALL, such as thrombocytopenia, neutropenia, infection, bleeding events, or corticosteroid-related bone fragility.

### 4.1. Applicability of Evidence

Regarding the applicability of the evidence, the included studies suggest that exercise-based interventions with aerobic components may be feasible and potentially beneficial for pediatric patients with ALL across different stages of treatment and survivorship. The interventions were implemented in both supervised clinical settings and home-based environments, indicating that exercise programs may be adaptable to different healthcare contexts and levels of resource availability. Furthermore, most interventions incorporated relatively accessible activities such as walking, running, stretching, strengthening exercises, cycling, and play-based aerobic activities, which may facilitate implementation in pediatric rehabilitation settings.

However, several factors may limit the generalizability and applicability of the findings. First, the included studies presented considerable heterogeneity regarding participant characteristics, treatment phases, intervention protocols, duration, intensity, and outcome measures. In addition, sample sizes were relatively small, and most studies were conducted in single-center settings, potentially limiting external validity. The interventions also varied between supervised and home-based approaches, which may influence adherence, monitoring, and reproducibility in routine clinical practice.

Another important consideration is that most participants were clinically stable and physically able to participate in exercise interventions. Therefore, the applicability of these findings to children with more severe treatment-related complications, greater functional impairment, or acute clinical instability remains uncertain. Moreover, safety monitoring and adverse event reporting were inconsistently described across studies, limiting conclusions regarding the implementation of exercise programs in higher-risk pediatric oncology populations.

The applicability of these findings to the broader pediatric oncology population requires caution. Children with different cancer diagnoses may share rehabilitation needs, including reduced endurance, muscle weakness, treatment-related fatigue, and limitations in mobility and activities of daily living. Accordingly, the exercise principles represented in the included ALL studies—particularly the use of structured, progressive, and individually adapted interventions incorporating aerobic components—may provide a relevant rehabilitation framework for other pediatric oncology populations. However, the magnitude and certainty of the effects observed in this review cannot be assumed to apply to other pediatric malignancies. Differences in disease biology, treatment regimens and cumulative treatment exposure, hematologic and cardiopulmonary toxicities, musculoskeletal complications, treatment phase, and baseline functional status may influence exercise tolerance, safety, feasibility, and responsiveness to rehabilitation. Therefore, the present findings should be considered directly applicable to children and adolescents with ALL, while their extension to other pediatric cancers should be regarded as hypothesis-generating and evaluated in diagnosis-specific studies.

Overall, the available evidence suggests that structured exercise interventions may be applicable as supportive rehabilitation strategies for selected pediatric patients with ALL. Nevertheless, the limited methodological quality, small sample sizes, and heterogeneity among studies indicate that the applicability of the evidence should be interpreted cautiously, and individualized clinical decision-making remains necessary.

### 4.2. Quality of Evidence

Overall, the quality of the evidence was limited by methodological concerns identified across the included studies. Four trials did not clearly report the methods used for random sequence generation or allocation concealment, raising concerns regarding potential selection bias [14,15,23,24]. Attempts to verify additional methodological details through published protocols or trial registrations were unsuccessful, particularly for the older studies.

Blinding procedures were poorly described across all included studies. Given the nature of exercise-based interventions, complete blinding of participants and personnel may not have been feasible; however, none of the trials clearly reported strategies to minimize performance or detection bias. In particular, the absence of information regarding blinded outcome assessments raises concerns, especially for studies using self-reported or parent-reported quality-of-life measures. The use of subjective outcome instruments may have increased susceptibility to expectancy effects and reporting bias.

Additional methodological limitations included small sample sizes, single-center designs, heterogeneity of intervention protocols, and variability in outcome measures. These factors contributed to imprecision and limited comparability between studies. Furthermore, most studies did not report pre-registered protocols or published statistical analysis plans, making it difficult to determine whether all intended outcomes were measured and reported transparently. This raises the possibility of selective reporting bias.

Despite these limitations, the available evidence suggests that exercise interventions containing aerobic components may be feasible and may contribute to improvements in physical functioning among children and adolescents with ALL. However, the certainty surrounding these effects remains limited, particularly for quality-of-life outcomes. Consequently, the observed benefits should be interpreted cautiously until confirmed by adequately powered randomized controlled trials employing rigorous methodology, standardized intervention protocols, blinded outcome assessment where feasible, and consistent reporting of clinically relevant outcomes.

### 4.3. Possible Biases in the Review Process

This review adhered to the methodological standards of the Cochrane Handbook. Screening and data extraction were conducted independently and in duplicate to minimize selection bias. The original bibliographic search included MEDLINE, Scopus, and Web of Science and was subsequently supplemented by searches of CENTRAL, ClinicalTrials.gov, and WHO ICTRP, together with reference-list screening of included studies and relevant systematic reviews. Although these additional searches increased coverage of published, registered, ongoing, and potentially unpublished trials, the possibility that relevant studies were missed cannot be completely excluded, particularly because dedicated searches of conference proceedings and other gray-literature sources were not performed.

### 4.4. Agreements or Disagreements with Other Studies or Reviews

A growing body of evidence supports the role of aerobic and multimodal exercise interventions in improving physical and psychosocial outcomes among patients with hematologic malignancies, particularly in adult populations. Systematic reviews and meta-analyses conducted in adults with leukemia and other hematologic cancers have consistently reported beneficial effects of aerobic exercise on cardiorespiratory fitness, fatigue, functional capacity, and quality of life. For example, Knips et al. [25], in a Cochrane systematic review, demonstrated that structured aerobic exercise programs are associated with improvements in physical fitness and reductions in cancer-related fatigue in adults undergoing or following treatment for hematologic malignancies. These findings provide a relevant conceptual framework supporting the potential benefits of exercise-based interventions in pediatric oncology populations.

However, extrapolation of evidence from adult populations to children and adolescents must be approached with caution, given fundamental differences in disease biology, treatment protocols, developmental stages, and outcome priorities. In this context, the present review contributes novel and clinically relevant evidence by focusing exclusively on pediatric patients with acute lymphoblastic leukemia and by restricting inclusion to randomized controlled trials evaluating exercise interventions incorporating aerobic components.

More recently, Liu et al. [9] conducted a systematic review with meta-analysis examining the effects of physical exercise interventions in children with ALL during the consolidation phase of treatment and rehabilitation. In contrast to our review, their outcomes encompassed bone mineral density, cardiorespiratory fitness, joint range of motion, muscle strength, functional mobility, cancer-related fatigue, and quality of life. Quality of life was assessed in four randomized controlled trials using the PedsQL, overlapping with the primary outcome of our review.

The findings of Liu et al. suggest that long-term, regular, multimodal exercise interventions may be more effective than aerobic exercise alone in improving quality of life and cardiorespiratory fitness in pediatric patients with ALL. These results are partially consistent with the findings of our review, particularly regarding improvements in functional mobility and functional exercise capacity. However, the broader scope and inclusion of heterogeneous exercise modalities and study designs in their meta-analysis limit direct comparability with our findings.

An earlier systematic review by Coombs et al. [6] evaluated the effects of exercise and motor interventions on physical activity levels and motor outcomes in children with ALL during and after chemotherapy. This review included 19 studies (11 randomized controlled trials, 2 controlled clinical trials, and 6 cohort studies) involving 508 children with ALL and summarized outcomes separately by treatment phase. In contrast, our review exclusively included randomized controlled trials, as this study design provides the highest level of evidence for assessing intervention effectiveness.

Importantly, Coombs et al. [6] identified substantial methodological limitations across included studies, with many trials rated as having moderate-to-high risk of bias due to inadequate allocation concealment, poor adherence reporting, and selective outcome reporting. These concerns align with the high risk of bias identified in the studies included in our review and reinforce the conclusion that the overall certainty of evidence supporting exercise interventions in pediatric ALL remains low.

Taken together, the existing adult and pediatric literature supports the potential role of exercise interventions in hematologic malignancies. Nevertheless, the present review offers an updated and focused synthesis of randomized evidence specifically addressing exercise with aerobic components in pediatric ALL, highlighting persistent gaps in high-quality trial design, outcome standardization, and long-term follow-up. These findings underscore the need for rigorously designed randomized controlled trials to better define the role of aerobic exercise within pediatric oncology rehabilitation protocols.

## 5. Conclusions

### 5.1. Implications for Practice

The evidence regarding the effects of exercise interventions with aerobic components on quality of life in children and adolescents with ALL remains very uncertain. Low-certainty evidence suggests that these interventions may improve functional mobility and functional exercise capacity/endurance at the medium term (3–6 months). The benefits cannot be attributed specifically to the aerobic component of predominantly multimodal interventions. Additional well-designed randomized controlled trials are needed to strengthen confidence in these findings.

### 5.2. Implications for Research

The included studies presented several methodological limitations that reduced the certainty of the evidence. Common concerns included insufficient reporting of randomization and allocation concealment procedures, lack of blinding of participants, caregivers, and outcome assessors, absence of pre-registered protocols, and incomplete reporting of adverse event monitoring. Furthermore, quality-of-life outcomes were frequently assessed using self-reported or parent-reported instruments, increasing susceptibility to detection bias.

Additional limitations included small sample sizes, single-center designs, heterogeneity of intervention protocols, variability in treatment phases, and inconsistent outcome measures, which limited comparability between studies and reduced the precision of the findings.

Future randomized controlled trials should aim to: implement and clearly report appropriate randomization and allocation concealment methods; incorporate blinded outcome assessment whenever feasible; prospectively register study protocols and statistical analysis plans; standardize intervention protocols and outcome measures; include standardized safety monitoring procedures and predefined adverse event reporting criteria to better characterize the safety of exercise interventions; include larger and more diverse samples; report adherence, safety monitoring, and adverse events more comprehensively; and evaluate long-term effects of exercise interventions through extended follow-up periods. Addressing these limitations would strengthen the quality, reproducibility, and clinical applicability of the evidence regarding exercise-based rehabilitation in pediatric patients with ALL.

## Figures and Tables

**Table 1:** General description of the selected studies.

Study	Participant Characteristics			Objectives	Intervention	Outcomes
	Country	Population	Age			
Elnaggar et al. (2025) [19]	Saudi Arabia	58 pediatric patients with ALL post-treatment (60.34% male)	13.8 ± 2.47 years	Identify the benefits of aerobic exercise with combined traditional rehabilitation for ALL survivors.	Twelve-week supervised dose-graded aerobic exercise program (3 sessions/week) combined with traditional physical rehabilitation, with progressive increases in exercise intensity and duration.	Cardiopulmonary fitness outcomes included VO2peak, VEq/VO2, VE, O2P, HRmax, HRR1, and RER. Physical performance outcomes included 6MWT, TUDS, and 4 × 10 m shuttle run test.
Khodashenas et al. (2017) [23]	Iran	20 children with ALL undergoing chemotherapy (60% male)	9.5 years (range 5–12 years)	Evaluate the effectiveness of an aerobic exercise program on quality of life in children undergoing cancer treatment.	Twelve-week aerobic exercise program including walking, running, and different forms of play; 3 sessions/week, 60 min/session, at 60–85% of maximum heart rate, in addition to routine treatment.	Quality of life assessed using PedsQL version 4. Significant improvements were observed mainly inpain and hurt (reported as pain/injury) and cognitive problem subscales, particularly according to parent reports.
Tanir & Kuguoglu (2013) [14]	Turkey	41 children with ALL initially randomized; 40 completed the study (60.0% male among completers).	10.5 years (range 8–12 years)	Determine the effects of an exercise program on physical parameters and quality of life in children with ALL.	Three-month home-based exercise program including active ROM exercises, muscle strengthening, and aerobic exercise (walking, cycling, rope jumping, dancing, slow running).	9 min walk test, stair climbing, TUG, muscle strength, hemoglobin and hematocrit values; quality of life assessed using PedsQL 3.0 and 4.0.
Marchese et al. (2004) [15]	United States	28 children with ALL receiving maintenance chemotherapy (71.4% male)	7.7 years (range 4–15 years)	Examine the effects of physical therapy intervention in children with ALL.	Individualized 4-month physical therapy program including ankle dorsiflexion stretching, lower-extremity strengthening, aerobic exercise, and home-based exercises with family consultation.	ROM, muscle strength, TUDS, 9 min run–walk test, and quality of life assessed with PedsQL 3.0.
Cox et al. (2018) [24]	United States	107 children and adolescents with newly diagnosed ALL (65.4% male)	4–18 years	To evaluate the effects of a home-based physical activity intervention combined with motivational support on physical performance, quality of life, and physical activity levels in children with ALL.	Participants received a physical activity intervention consisting of routine physical therapy exercises targeting strength, range of motion, gross motor skills, and endurance, combined with motivational strategies. Exercises were prescribed for approximately 30 min/session, 5 times/week over 24 months.	Quality of life, physical activity, 6-Minute Walk Test, BOT-2 motor proficiency, ankle range of motion, muscle strength.

**Abbreviations:** ALL: acute lymphoblastic leukemia; VO2peak: peak oxygen consumption; VEq/VO2: ventilatory equivalent for oxygen; VE: minute ventilation; O2P: oxygen pulse; HRmax: maximum heart rate; HRR1: one-minute heart rate recovery; RER: respiratory exchange ratio; 6MWT: 6-Minute Walk Test; TUDS: Timed Up and Down Stairs; PedsQL: Pediatric Quality of Life Inventory; ROM: range of motion; TUG: Timed Up and Go test; BOT-2: Bruininks–Oseretsky Test of Motor Proficiency, Second Edition. **Note:** Participant counts refer to the initial study sample unless otherwise specified. In Tanir and Kuguoglu, sex-distribution data refer to the 40 participants who completed the study. Numbers contributing to individual outcome analyses varied.

**Table 2:** Summary of findings: Effects of exercise interventions containing aerobic components on functional mobility, functional capacity, and quality of life in children and adolescents with ALL.

Outcomes	Effect Estimate (95% CI)	No. of Participants (Trials)	Certainty of the Evidence (GRADE)	Comments
Functional mobility at medium term (3–6 months)	Exercise interventions may improve functional mobility compared with control interventions, MD −1.03 s (95% CI −1.99 to −0.08)	126 participants (3)	⊕⊕⊝⊝ low ^a,b^	Exercise interventions may improve functional mobility in children and adolescents with ALL, as measured by the Timed Up and Down Stairs test at medium term (3–6 months)
Functional capacity at medium term (3–6 months)	The pooled effect favored exercise interventions: SMD (Hedges’ g) = 0.60 (95% CI 0.25 to 0.96)	126 participants (3)	⊕⊕⊝⊝ low ^a,b^	Exercise interventions may improve functional capacity in children and adolescents with ALL at medium term (3–6 months)
Quality of life	Data were insufficient for quantitative synthesis	165 participants (4)	⊕⊝⊝⊝ very low ^a,c,d^	The evidence is very uncertain about the effect of exercise on quality of life in children and adolescents with ALL

Abbreviations: CI: confidence interval; MD: mean difference; SMD: standardized mean difference; ALL: acute lymphoblastic leukemia; GRADE: Grading of Recommendations Assessment, Development and Evaluation; RoB 2: Cochrane risk-of-bias tool for randomized trials, version 2. **GRADE Working Group grades of evidence**. **High certainty**: We are very confident that the true effect lies close to that of the estimate of the effect. **Moderate certainty**: We are moderately confident in the effect estimate: The true effect is likely to be close to the estimate of the effect, but there is a possibility that it is substantially different. **Low certainty**: Our confidence in the effect estimate is limited: The true effect may be substantially different from the estimate of the effect. **Very low certainty**: We have very little confidence in the effect estimate: The true effect is likely to be substantially different from the estimate of effect. ^a^ Downgraded one level for risk of bias because, for functional mobility and functional capacity, two of the three contributing trials were judged at high risk of bias and one presented some concerns, and, for quality of life, all four contributing trials were judged at high risk of bias (RoB 2). ^b^ Downgraded one level for imprecision due to the small overall sample size and limited number of studies. ^c^ Downgraded one level for inconsistency because quality-of-life outcomes were assessed using different instruments and reporting methods, with heterogeneous findings across studies. ^d^ Downgraded one level for imprecision because of small sample sizes and insufficient numerical data for quantitative synthesis.

## Data Availability

No new data were created or analyzed in this study. Data sharing is not applicable to this article.

## Supplementary Materials

The following supporting information can be downloaded at: https://www.mdpi.com/article/10.3390/cancers18213454/s1, Figure S1: Sensitivity analysis of functional exercise capacity/endurance excluding Marchese et al. [15]; File S1: PRISMA 2020 checklist.

## Abbreviations

The following abbreviations are used in this manuscript:

ALL	Acute lymphoblastic leukemia
BOT-2	Bruininks–Oseretsky Test of Motor Proficiency, Second Edition
CENTRAL	Cochrane Central Register of Controlled Trials
CI	Confidence interval
CHQ	Child Health Questionnaire
GRADE	Grading of Recommendations Assessment, Development and Evaluation
HRmax	Maximum heart rate
HRR1	One-minute heart rate recovery
MD	Mean difference
MeSH	Medical Subject Headings
O_2_P	Oxygen pulse
PedsQL	Pediatric Quality of Life Inventory
PRISMA	Preferred Reporting Items for Systematic Reviews and Meta-Analyses
PROSPERO	International Prospective Register of Systematic Reviews
RCT	Randomized controlled trial
REML	Restricted maximum likelihood
RER	Respiratory exchange ratio
RoB 2	Risk of Bias 2 tool
ROM	Range of motion
SD	Standard deviation
SMD	Standardized mean difference
SoF	Summary of Findings
TUDS	Timed Up and Down Stairs test
TUG	Timed Up and Go test
VE	Minute ventilation
VEq/VO_2_	Ventilatory equivalent for oxygen
VO_2_peak	Peak oxygen consumption
WHO ICTRP	World Health Organization International Clinical Trials Registry Platform
6MWT	Six-Minute Walk Test

## References

[1] Aureli A., Marziani B., Venditti A., Sconocchia T., Sconocchia G. (2023). Acute lymphoblastic leukemia immunotherapy treatment: Now, next, and beyond. Cancers.

[2] Inaba H., Greaves M., Mullighan C.G. (2013). Acute lymphoblastic leukaemia. Lancet.

[3] PDQ Adult Treatment Editorial Board (2002). Acute lymphoblastic leukemia treatment (PDQ^®^): Health professional version. PDQ Cancer Information Summaries [Internet].

[4] National Cancer Institute Cancer in Children and Adolescents. https://www.cancer.gov/types/childhood-cancers/child-adolescent-cancers-fact-sheet.

[5] Braam K.I., van der Torre P., Takken T., Veening M.A., van Dulmen-den Broeder E., Kaspers G.J.L. (2016). Physical exercise training interventions for children and young adults during and after treatment for childhood cancer. Cochrane Database Syst. Rev..

[6] Coombs A., Schilperoort H., Sargent B. (2020). The effect of exercise and motor interventions on physical activity and motor outcomes during and after medical intervention for children and adolescents with acute lymphoblastic leukemia: A systematic review. Crit. Rev. Oncol. Hematol..

[7] American College of Sports Medicine (2021). ACSM’s Guidelines for Exercise Testing and Prescription.

[8] Riggs L., Piscione J., Laughlin S., Cunningham T., Timmons B.W., Courneya K.S., Bartels U., Skocic J., de Medeiros C., Liu F. (2017). Exercise training for neural recovery in a restricted sample of pediatric brain tumor survivors: A controlled clinical trial with crossover of training versus no training. Neuro-Oncol..

[9] Liu H., Yin J., Wang K., Liu S., Yang Y., Song Z., Dong C., Zhang T., Luo J. (2024). Efficacy of physical exercise intervention on children with acute lymphoblastic leukemia during treatment and rehabilitation: A systematic review and meta-analysis. Support. Care Cancer.

[10] Page M.J., McKenzie J.E., Bossuyt P.M., Boutron I., Hoffmann T.C., Mulrow C.D., Shamseer L., Tetzlaff J.M., Akl E.A., Brennan S.E. (2021). The PRISMA 2020 statement: An updated guideline for reporting systematic reviews. BMJ.

[11] Higgins J.P.T., Thomas J., Chandler J., Cumpston M., Li T., Page M.J. (2021). Cochrane Handbook for Systematic Reviews of Interventions Version 6.1.

[12] Varni J.W., Burwinkle T.M., Katz E.R., Meeske K., Dickinson P. (2002). The PedsQL™ in pediatric cancer: Reliability and validity of the Pediatric Quality of Life Inventory generic core scales, multidimensional fatigue scale, and cancer module. Cancer.

[13] Li A.M., Yin J., Yu C.C.W., Tsang T., So H.K., Wong E., Chan D., Hon E.K.L., Sung R. (2005). The six-minute walk test in healthy children: Reliability and validity. Eur. Respir. J..

[14] Tanir M.K., Kuguoglu S. (2013). Impact of exercise on lower activity levels in children with acute lymphoblastic leukemia: A randomized controlled trial from Turkey. Rehabil. Nurs..

[15] Marchese V.G., Chiarello L.A., Lange B.J. (2004). Effects of physical therapy intervention for children with acute lymphoblastic leukemia. Pediatr. Blood Cancer.

[16] Gocha-Marchese V.G., Chiarello L.A., Lange B.J. (2003). Strength and functional mobility in children with acute lymphoblastic leukemia. Med. Pediatr. Oncol..

[17] Zaino C.A., Marchese V.G., Westcott S.L. (2004). Timed up and down stairs test: Preliminary reliability and validity of a new measure of functional mobility. Pediatr. Phys. Ther..

[18] Sterne J.A.C., Savović J., Page M.J., Elbers R.G., Blencowe N.S., Boutron I., Cates C.J., Cheng H.Y., Corbett M.S., Eldridge S.M. (2019). RoB 2: A revised tool for assessing risk of bias in randomised trials. BMJ.

[19] Elnaggar R.K., Osailan A.M., Elbanna M.F., Abd-Elmonem A.M. (2025). Effectiveness of a dose-graded aerobic exercise regimen on cardiopulmonary fitness and physical performance in pediatric survivors of acute lymphoblastic leukemia: A randomized clinical trial. J. Cancer Surviv..

[20] Deeks J.J., Higgins J.P.T., Altman D.G., Higgins J.P.T., Thomas J., Chandler J., Cumpston M., Li T., Page M.J., Welch V.A. (2023). Chapter 10: Analysing data and undertaking meta-analyses. Cochrane Handbook for Systematic Reviews of Interventions Version 6.4.

[21] Cohen J. (1988). Statistical Power Analysis for the Behavioral Sciences.

[22] Santesso N., Glenton C., Dahm P., Garner P., Akl E.A., Alper B., Brignardello-Petersen R., Carrasco-Labra A., De Beer H., Hultcrantz M. (2020). GRADE guidelines 26: Informative statements to communicate the findings of systematic reviews of interventions. J. Clin. Epidemiol..

[23] Khodashenas E., Badiee Z., Sohrabi M., Ghassemi A., Hosseinzade V. (2017). The effect of an aerobic exercise program on the quality of life in children with cancer. Turk. J. Pediatr..

[24] Cox C.L., Zhu L., Kaste S.C., Srivastava K., Barnes L., Nathan P.C., Wells R.J., Ness K.K. (2018). Modifying bone mineral density, physical function, and quality of life in children with acute lymphoblastic leukemia. Pediatr. Blood Cancer.

[25] Knips L., Bergenthal N., Streckmann F., Monsef I., Elter T., Skoetz N. (2019). Aerobic physical exercise for adult patients with haematological malignancies. Cochrane Database Syst. Rev..

