# Supplementary material for: Exercise Interventions Incorporating Aerobic Components in Children and Adolescents with Acute Lymphoblastic Leukemia: A Systematic Review and Meta-Analysis
Source: Cancers (Basel). 2026 Oct 26;18(21):3454. doi: 10.3390/cancers18213454 (PMC13762348; doi:10.3390/cancers18213454)
Supplement: Supplementary file 1 [file cancers-18-03454-s001.zip › Supplementary Figure S1.pdf]

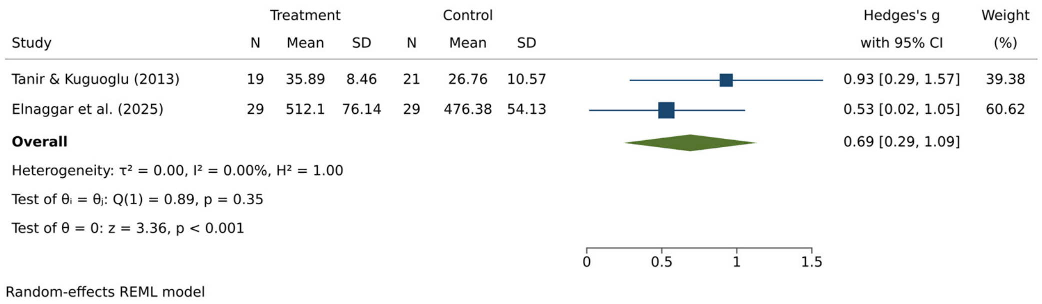

Supplementary Figure S1. Sensitivity analysis of functional exercise capacity/endurance excluding Marchese et al., who used a nine-minute run–walk test. The analysis included walking tests reported by Tanir and Elnaggar. Effects were pooled using a random-effects restricted maximum likelihood (REML) model. Positive Hedges’ g values favor exercise; Tanir and Kuguoglu (2013) [14]; Elnaggar et al. (2025) [19].
